# Mouse primary follicles experience slow growth rates after activation and progressive increases that influence the duration of the primary follicle phase^†^

**DOI:** 10.1093/biolre/ioad095

**Published:** 2023-08-08

**Authors:** Sharon Richard, Nicholas J Anderson, Yiran Zhou, Michael W Pankhurst

**Affiliations:** Department of Anatomy, School of Biomedical Sciences, University of Otago, Dunedin, New Zealand; Department of Anatomy, School of Biomedical Sciences, University of Otago, Dunedin, New Zealand; Department of Anatomy, School of Biomedical Sciences, University of Otago, Dunedin, New Zealand; Department of Anatomy, School of Biomedical Sciences, University of Otago, Dunedin, New Zealand

**Keywords:** ovarian follicle, granulosa cell, proliferation, 5-bromo-2′-deoxyuridine, folliculogenesis

## Abstract

There are conflicting estimates of the duration of mouse primary follicle development. An accurate determination is needed for studies examining preantral follicle survival and mathematical modeling of folliculogenesis. Primary follicle granulosa cell proliferation rates are low and variable, which may explain the variation in duration estimates. In the present study, female C57Bl6/J mice were exposed to bromodeoxyuridine for 48 hours, to label the proliferating granulosa cells in a large proportion of primary follicles. The bromodeoxyuridine-containing water was then withdrawn and replaced with drug-free water and the mice were euthanized at 0, 1, 3, 6, 10, or 13 days post-bromodeoxyuridine withdrawal. Granulosa cells were bromodeoxyuridine labeled in 48% of primary follicles at day 0, but this decreased to 5% over the 13-day period, as the labeled primary follicles progressed to the secondary follicle stage. Curve-fitting estimated that the last of the bromodeoxyuridine-labeled primary follicles would progress to the secondary stage by 13.7 days. Mathematical models that assumed constant rates of primary follicle proliferation were fitted to the data, but the observed pattern of bromodeoxyuridine-labeled primary follicle disappearance could not be replicated. The level of immunoreactivity for bromodeoxyuridine and proliferating-cell nuclear antigen in primary follicles revealed follicles with no granulosa cell proliferation during the 48-h bromodeoxyuridine-exposure period had resumed proliferation 1 or 3 days later. Therefore, primary follicle granulosa cells proliferate after follicle activation, but proliferation rates gradually increase as the follicle develops. Prior estimates of primary follicle duration are inaccurate due to the assumption that follicles develop at a constant rate.

## Teaser

The length of the primary follicle phase is 14–16 days in mice but follicle development pauses briefly, shortly after primordial follicle activation, and explains why prior mathematical model-based estimates were inaccurate.

## Introduction

The duration of ovarian follicle development in rodents has been estimated using a variety of methods. Developmental milestones have been used to provide estimates in mice, with the primary follicles appearing at postnatal days 2–4 and secondary follicles emerging at day 5 [1, 2]. However, studies using radioactive labeling of proliferating granulosa cells and mathematical modeling in adult mice have estimated the duration of the primary follicle phase to be as short as 7 days [3, 4] or as long as 9–17 days [5]. However, the former studies [3, 4] were conducted before the discovery of the G0 phase of the cell cycle [6] and relied on formulae that involve the S-phase duration, calculated with methods assuming that granulosa cells constantly proliferate without entering G0. The latter study [5] used a tritiated thymidine pulse-chase approach that showed the loss of radiolabeled primary follicles as they progressed to the secondary stage, but the 8-day period between the last two timepoints in the study (9 and 17 days) prevented accurate determination of the duration of primary follicle development.

Recently, a transgenic-reporter gene technique was used to estimate an interval of 7 days between the primordial and primary follicle phases, but the point of division between these two follicle stages was not precisely defined [7]. Primary and secondary follicles are distinguished by whether they have one or two layers of granulosa cells, respectively, but there is no consensus on whether the follicle reaches the secondary stage, when the follicle attains its first granulosa cell in the second layer or at the point when the second layer becomes contiguous around the entire follicle. More recent studies have used prolonged AMH treatment to inhibit primordial follicle activation and deplete the ovary of preantral and antral follicles, demonstrating that the first secondary follicles reappeared 5–10 days after AMH withdrawal [8, 9]. However, AMH only partially inhibits primordial follicle activation and the loss of the remaining developing follicles is likely caused by atresia late in the primary follicle stage [10]. Therefore, the 5–10 days probably only represents the latter part of primary follicle development. Currently, there is a lack of consensus on the duration of the primary phase of follicle development in mice.

Developing follicles in the prepubertal mouse are derived from a distinct population that develops at a faster rate than primary follicles in adulthood [7] indicating that experiments to determine adult primary follicle duration must be performed in adult mice. An early primary follicle can have as few as 6 pre-granulosa cells which increases to ~70 granulosa cells at the end of the primary phase. This requires that each granulosa cell undergoes three to four rounds of mitosis to reach the end of the primary stage. Proliferating granulosa cells are rarely observed in primary follicles in adulthood [11], which is consistent with the process being protracted. It is not clear if this occurs because the granulosa cells have prolonged periods of non-proliferation (G0 phase) or if one or more of stages of cell division are prolonged during this stage of folliculogenesis. This uncertainty affects experimental design and interpretation, and the accuracy of mathematical models, when studying folliculogenesis.

This study used bromodeoxyuridine (BrdU), a synthetic thymidine analogue that is incorporated into the DNA during the S-phase of the cell cycle, to perform pulse-chase experiments to determine the duration of the mouse primary follicle phase. BrdU was supplied to the animals in their drinking water for 48 h (pulse period), and animals were euthanized at various intervals after withdrawal (chase period). The time taken for the last of the primary follicles that had incorporated BrdU to transition to the secondary phase was deemed to represent the duration of the primary follicle phase. This approach was chosen as it can determine the duration of the primary follicle phase without prior knowledge of granulosa cell proliferation speeds.

## Methods

### Animals

Female C57Bl6/J mice (42–100 days old) were housed in individually ventilated cages in a temperature- and humidity-controlled facility with ad libitum access to food and water, under 12:12 h light/dark cycles. All experiments were approved by the University of Otago Animal Ethics Committee.

### Experiment design

**Figure 1:**
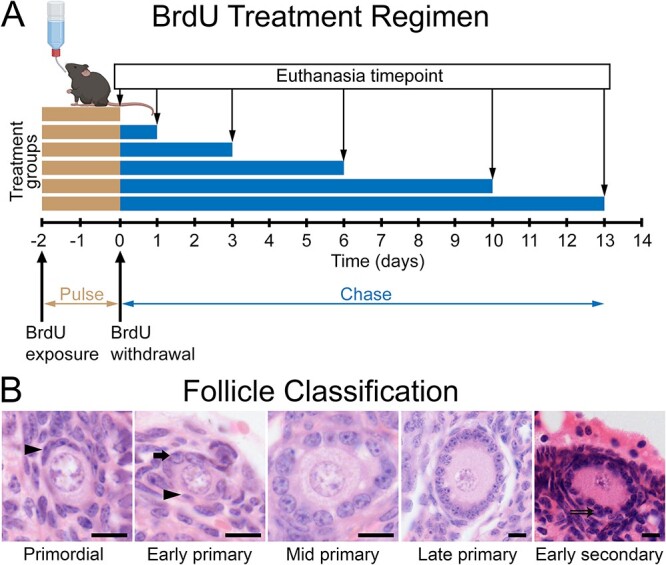
BrdU treatment regimen and follicle classification criteria. (**A**) Mice were exposed to BrdU in their drinking water for 48 h. The BrdU-containing drinking water was then replaced with compound-free drinking water (time = zero). Groups of mice (*n* = 3) were either euthanized at 0, 1, 3, 6, 10, or 13 days after BrdU withdrawal and the ovaries were examined by immunohistochemistry for BrdU. (**B**) Follicles were classified as primordial when all surrounding pre-granulosa cells had flattened morphology (arrowhead). Follicles were classified as primary from the first appearance of any cuboidal granulosa cells (early primary) surrounding the oocyte (some flattened pre-granulosa cells may still be present) and including all large follicles with a single continuous layer of cuboidal granulosa cells (mid and late primary). The end of the primary phase/beginning of the secondary phase was when at least two layers of granulosa cells were present at any point surrounding the oocyte (double arrowhead). Scale bars: 10 μm.

Mice (*n* = 3 per timepoint) were supplied with drinking water containing 1 mg/mL BrdU (5-bromo-2′-deoxyuridine; Merck, cat# B5002) for 48 h beginning at 10:00 (Figure 1A). The water bottle was replaced with freshly prepared BrdU-containing drinking water after 24 h. Water bottle weight was recorded every 24 h. At the end of the 48-h BrdU exposure period, BrdU-containing drinking water was removed and replaced with fresh water containing no additives. The mean (±SD) water intake per mouse was 4.2 ± 0.4 mL per day during the BrdU treatment and 4.0 ± 0.5 mL per day during the withdrawal period (*P* = 0.369, Student’s *t*-test). The time that the BrdU was withdrawn was designated time = zero days, and animals were euthanized at 0-, 1-, 3-, 6-, 10-, and 13-day timepoints (Figure 1A). Ovaries were dissected from the mice and one from each animal was immersion fixed in Bouins’ fixative (picric acid 0.9% w/v, formaldehyde 8% w/v, glacial acetic acid 5% w/v). Ovaries were embedded in paraffin wax and 5-μm-thick sections were mounted onto microscope slides.

### Immunohistochemistry

Five 5-μm-thick sections from one ovary of each mouse were examined for BrdU labeling and quantification in primary follicles. Each of the tissue sections examined was separated by at least 50 μm within the tissue sectioning series to avoid double-counting of primary follicles in adjacent sections. The sections were dewaxed in three changes of xylene and were rehydrated in graded ethanol washes (100%, 100%, 95%, and 70% v/v). Antigenic sites were exposed by a 60-min immersion in 1 M hydrochloric acid at room temperature followed by neutralization in 0.1 M sodium borate buffer for 10 min. The sections were washed with 0.01 M phosphate-buffered saline (PBS) and blocked with 1:20 dilution of normal donkey serum (Merck, cat# D9663, RRID: AB_2810235) diluted 1:20 in PBS, with 0.2% Tween 20 (Merck, cat# P1379) for 20 min. Primary antibody (rat anti-BrdU, Abcam, cat#: ab6326, RRID: AB_305426) was applied at 2 μg/mL PBS with 0.2% w/v Tween 20 (Sigma-Aldrich, Cat# P1379) and 1% w/v bovine serum albumin overnight at 4°C. Secondary antibodies (3 μg/mL biotinylated donkey anti-rat IgG, Jackson ImmunoResearch, cat# 712-065-150, RRID: AB_2340646) and streptavidin–biotin–HRP complex (1:200, Amersham, RPN1051) were each applied for 1 h at room temperature. Immunoreactivity was visualized with diaminobenzidine (DAB substrate kit; Vector Labs, cat# SK-4100) and the sections were counterstained with hematoxylin and eosin.

Proliferating-cell nuclear antigen (PCNA) immunohistochemistry was performed in adjacent sections to BrdU-labeled tissue, with the omission of the hydrochloric and sodium borate steps, as these steps abolished immunoreactivity. Instead, microwave antigen retrieval was performed in citrate buffer, pH 6.0, but the remainder of the protocol was identical (primary antibody: 0.05 μg/mL mouse anti-PCNA, Santa Cruz Biotechnology, cat# sc-56; secondary antibody, RRID: AB_628110: 2.8 μg/mL, biotinylated donkey anti-mouse IgG, Jackson ImmunoResearch, cat# 715-065-151, RRID: AB_2340785).

The specificity of the immunohistochemical technique was confirmed by substituting the primary antibody with IgG isolated from non-immune serum from the same species (mouse IgG, Merck, cat# 15381, RRID: AB_2827936; rat IgG, ThermoFisher Scientific, cat# 02-9602, RRID: AB_2532969). Non-specific immunoreactivity was not observed in the ovary in these control experiments.

### Morphological classification and follicle counting

All primordial, primary, and secondary follicles in the sampled sections (15 sections per timepoint, 5 sections per mouse) were examined. The number of BrdU immuno-positive (BrdU^+^) and immuno-negative (BrdU^−^) were quantified in each follicle and oocyte and total follicle diameter measurements were taken from microscope images using FIJI image-analysis software [12]. Follicle classification criteria are shown in Figure 1B. Follicle labeling index was calculated as number of BrdU^+^ cells divided by the total number of granulosa cells in the histological cross-section.

### Confocal microscopy

Immunohistochemistry for PCNA and BrdU was conducted as above, with the substitution of the following secondary antibodies: 1 μg/mL donkey anti-mouse DyLight 550 (ThermoFisher Scientific, cat# SA5-10167, RRID: AB_2556747), 3 μg/mL biotin-conjugated donkey anti-rat (Jackson ImmunoResearch, cat# 712-065-150, RRID: AB_2340646), and 2 μg/mL streptavidin-conjugated DyLight 650 (ThermoFisher Scientific, cat# 84547). Nuclei were labeled with DAPI (ThermoFisher Scientific, cat# D1306). Target primary follicles were located and scanned in the serial ovary sections (5 μm section thickness). Image stacks of each follicle were generated and aligned in ImageJ with the TrakEM2 alignment function. All granulosa cells were counted and scored as BrdU and PCNA positive or negative throughout the image stack to avoid double-counting granulosa cells that appeared in more than one section.

### Mathematical modeling

The number of granulosa cells in each primary follicle was estimated from cell counts in histological sections using the following formula [4]:


$$ N=n\left(\frac{V_1}{\frac{k}{f}{V}_2}\right) $$

where *N* = number of granulosa cells in the whole follicle, *n* = number of follicles visible in the largest cross-section, *V*_1_ = volume of the whole follicles, *V*_2_ = volume of the largest cross-section, *f* = section thickness, and *k* represents a correction factor, where *k* = 2*r*_c_ + *f* − 2*p* and *r*_c_ = radius of the nucleus of granulosa cells, *f* = section thickness, and *p* = height of the smallest segment of a granulosa cell which is visible.

The growth of primary follicles was modeled with two different formulae that assume constant rates of granulosa cell proliferation to determine if such models can accurately match the data observed in vivo. The first model was an iterative model that examines how many additional granulosa cells will be added to the follicle each day. Iterations of the model can be repeated for additional days of growth beyond the first iteration (day).


$$ {N}_{\mathrm{end}}={N}_{\mathrm{initial}}+\left(\frac{dT}{B}\right) LI\ {N}_{\mathrm{initial}} $$

where *N*_end_ = number of granulosa cells in the follicle cross-section at the end of the iteration, *N*_initial_ = number of granulosa cells in the follicle cross-section at the beginning of the iteration (0 h), *LI* = labeling index of granulosa cells within the follicle, *dT* = cell division time for a granulosa cell in hours, and *B* = BrdU exposure time in hours. Iterations were conducted with 12-, 24-, and 48-h cell-cycle durations such that each iteration represents the duration of one granulosa cell’s division to the next. These timepoints were chosen based on a wide range of estimates of this parameter in the literature [13, 14]. The formula adjusts the labeling index on the assumption that 48 h would be sufficient time for two or four rounds of granulosa cell mitosis, if the cell-cycle durations were 24 or 12 h, respectively. The *N*_initial_ values were obtained from the population of follicles observed at 0 h post-BrdU withdrawal (*N* = 248).

The second model used a formula previously used to calculate rates of granulosa cell proliferation [4].


$$ LI=\left(\exp \frac{t_s\ \ln\ 2}{T_{\mathrm{D}}}-1\right)\left(\exp \frac{t_2\ \ln\ 2}{T_{\mathrm{D}}}\right) $$

where *LI* = labeling index, *T*_D_ = doubling time, *t*_s_ = duration of the S-phase, and *t*_2_ = the G2 phase + half the duration of the mitosis phase. The values for *t*_s_ and *t*_2_ were obtained from Pedersen’s study [4] to calculate the granulosa cell doubling time in each follicle. Labeling indexes from the 0-h time point in the present study were used but had to be adjusted due to the more prolonged BrdU exposure period. The mean labeling index in the present study was 3.81 times higher than Pedersen’s study; thus, all labeling indexes were divided by 3.81. Primary follicle transition time (*T*_F_) for each primary follicle observed at the 0-h timepoint was calculated using the following formula [4]:


$$ {T}_{\mathrm{F}}={T}_{\mathrm{D}}\ \frac{\ln{N}_0-\ln{N}_i}{\ln 2} $$

where *N*_0_ = number of granulosa cells in the largest primary follicles (70 cells) and *N*_i_ = the number of granulosa cells in the smallest of primary follicles (6 cells).

### Analysis

Differences in BrdU- and PCNA-derived labeling index were calculated by subtracting the PCNA labeling index from the BrdU labeling index for each follicle. Differences between the 0-, 1-, and 3-day timepoints were analyzed by one-way ANOVA with Tukey’s *B* post hoc test.

## Results

**Figure 2:**
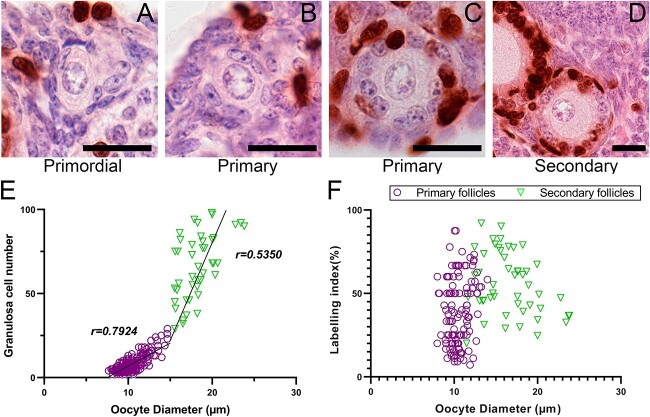
BrdU uptake over 48 h of exposure. Immunohistochemistry for BrdU (brown staining) at the day 0 timepoint in primordial (**A**), primary (**B**, **C**), and early secondary (**D**) follicles (scale bars: 20 μm). The relationships between granulosa cell number and oocyte diameter (**E**) and labeling index and oocyte diameter (**F**) are shown for primordial/primary (purple circles, *n* = 258) and secondary (green triangles, *n* = 34) follicles. BrdU: bromodeoxyuridine.

At the end of the 48-h BrdU exposure period (time = 0 days), no BrdU^+^ pre-granulosa cells were observed in any primordial follicles (*n* = 302, Figure 2A), suggesting that granulosa cells do not enter S-phase until after they have acquired cuboidal morphology. BrdU^+^ granulosa cells were observed in 48% of primary follicles (*n* = 248) with highly variable labeling indexes, ranging from 0 to 88%. Again, no granulosa cells with flattened morphology in transitioning follicles were observed to take up BrdU. All secondary follicles observed contained BrdU^+^ granulosa cells (median labeling index: 52%, range: 20–96%) and all non-atretic antral follicles had labeling indexes close to 100% (data not shown), indicating a progressive increase in granulosa cell proliferation rates with increasing follicle maturity. Correlation of oocyte diameter with granulosa cell counts (Figure 2F) demonstrated that these two variables have a close relationship in primary follicles (*r* = 0.79), with this relationship diminishing in secondary follicles (*r* = 0.53). No strong relationship was observed between oocyte diameter and labeling index (Figure 2F).

**Figure 3:**
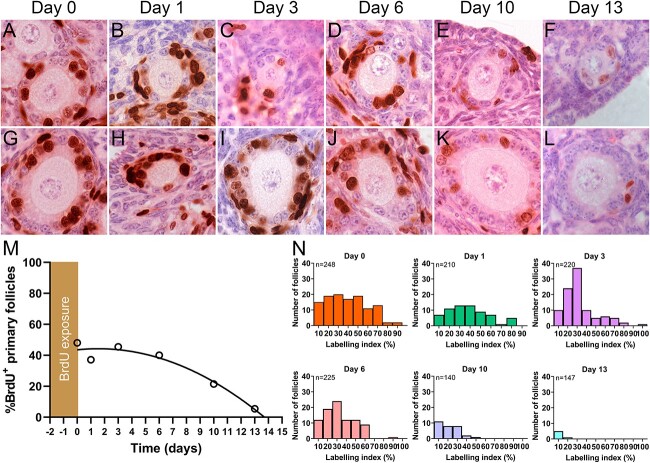
The progressive disappearance of BrdU-labeled primary follicles over time. BrdU^+^ granulosa cells were observed in primary follicles at all timepoints following BrdU withdrawal (**A**–**F**). Intensely labeled BrdU^+^ granulosa cells were present in secondary follicles at early post-BrdU withdrawal timepoints, with less-intense labeling at later timepoints (**G**–**L**). Scale bar: 20 μm. Declines in the percentage of BrdU^+^ primary follicles with time after BrdU withdrawal, fitted with quadratic equation data (**M**). Histograms demonstrate number of BrdU^+^ follicles remaining within each labeling index decile during the post-BrdU withdrawal period (**N**). BrdU: bromodeoxyuridine, *n*: total number of primary follicles counted including both BrdU^+^ and BrdU^−^.

The percentage of follicles containing BrdU^+^ granulosa cells was examined over 13 days after BrdU withdrawal to determine the time required for labeled primary follicles to progress to the secondary stage. The intensity of the BrdU staining within each granulosa cell decreased with increasing time in the BrdU withdrawal period, as the number of BrdU-containing chromosomes is reduced by half with each cell division (Figure 3A–F). However, it was expected that the labeling index during the 48-h exposure period would remain unchanged as the follicles continued to grow because the BrdU^+^ or BrdU^−^ status of each granulosa cell would be retained in the daughter cells after mitosis. Early secondary follicles containing BrdU^+^ granulosa cells were observed at all post-withdrawal timepoints indicating that BrdU^+^ primary follicles were progressing to the secondary stage over time (Figure 3G–L). At day 0, there were no secondary follicles with labeling indexes <25%. Secondary follicles with labeling indexes <25% were common at days 10 and 13, suggesting that they had arisen from follicles labeled with BrdU while still in the primary follicle stage. The mean labeling indexes in early secondary follicles at the 0-, 1-, 3-, 6-, 10-, and 13-day timepoints were 63, 56, 62, 30, 11, and 1%, respectively. The disappearance of BrdU^+^ primary follicles over time indicated that the time required for all labeled BrdU^+^ follicles to progress to the secondary stage was 13.7 days (Figure 3M).

The distribution of disappearance of BrdU^+^ follicles suggests that the follicles with the higher labeling indexes progress to the secondary stages earlier (Figure 3N). However, it was not clear why there was little change in the proportion of follicles containing BrdU^+^ granulosa cells in the first 6 days after BrdU withdrawal.

**Figure 4:**
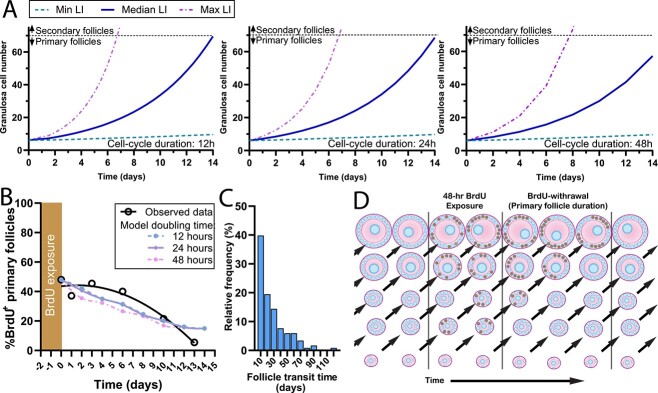
Modeling of primary follicle growth duration. (**A**) Primary follicle granulosa cell proliferation was modeled from the early primary stage (2 granulosa cells) until the secondary stage (≥26 granulosa cells). The minimum (7%), median (38%), and maximum (87%) labeling indexes (LI) from the day 0 data were used as variables in the model. (**B**) The proliferation of every BrdU^+^ primary follicle from day 0 dataset was used to model the progression of follicle development over time to show the percentage of BrdU^+^ follicles remaining. Follicles that crossed the 26 granulosa cell threshold at each timepoint were considered to be secondary follicles and were removed from the dataset. The rate of decline for the models with 12 h (blue dashed line), 24 h (purple solid line), and 48 h (pink dash-dot line) cell-cycle durations were all dissimilar to the observed data (black solid line). (**C**) Primary follicle transit times were calculated using the labeling indexes observed in all primary follicles at the zero-hour post-BrdU withdrawal timepoint based on the method of Pedersen et al. [4]. Data are displayed as a relative frequency distribution of the calculated transit time. (**D**) Schematic demonstrating the principle behind the mathematical models of BrdU-labeled primary follicle disappearance. BrdU is incorporated into proliferating granulosa cells in follicles at various stages of primary follicle development (brown nuclei). Follicles with sufficient proliferation to reach the secondary stage (>25 granulosa cells per cross-section) were removed from the BrdU^+^ contingent. The total number of follicles in the primary follicle pool remained constant because follicles progressing to the secondary phase were assumed to be replaced by ongoing primordial follicle activation. BrdU: bromodeoxyuridine, LI: labeling index.

The rate of loss of BrdU^+^ primary follicles in the post-withdrawal period did not appear to be consistent with a constant rate of granulosa cell proliferation. To investigate whether follicles with constant rates of development could generate the same pattern of follicle disappearance, two mathematical models were investigated. The first model was an iterative proliferation model used to generate follicle growth trajectories for the medium, maximum, or minimum observed proliferation rates in primary follicles (Figure 4A). The models demonstrate that follicles with median and maximal proliferation rates can transition to the secondary stage within 14 days, but the follicles with the lowest labeling indexes had very protracted predicted transition times. The transition times for follicles with the lowest LI, and assumed granulosa cell proliferation durations of either 12, 24, or 48 h, were 70, 71, and 90 days, respectively. Furthermore, applying these models to the data observed at the 0-h timepoint to determine the required time for each primary follicle to complete transition to the secondary stage was not able to replicate the observed pattern of follicle loss (Figure 4B). The modeling shows that the majority of primary follicles would need to increase their granulosa cell proliferation rates to match the data observed in vivo.

The previously described model [4] was used to generate follicle transition times for primary follicles based on the labeling indexes observed at the 0-h timepoint with the medium, maximum, or minimum proliferation rates observed with the BrdU staining (Figure 4C). This model predicts that more than one third of the labeled primary follicles would require >20 days to transition to the secondary follicle stage, which is not consistent with the observed pulse-chase data observed in Figure 3M. Both models required assumptions that had to be made regarding the duration of granulosa cell proliferation, but it is highly unlikely that any set of parameters in these models with constant rates of granulosa cell proliferation could generate results similar to those observed in vivo.

**Figure 5:**
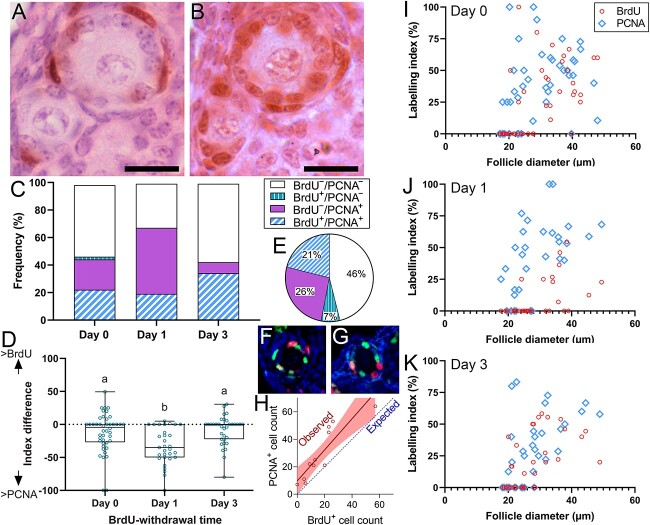
BrdU and PCNA labeling in adjacent sections of the same primary follicle. BrdU immunoreactivity represents proliferation that had occurred during the 48-h BrdU exposure period and PCNA immunoreactivity represents proliferation that was occurring at the time of euthanasia. Primary follicle granulosa cells that took up little or no BrdU label during the 48-h exposure period (**A**) were able to resume proliferation 24 h post-BrdU withdrawal (**B**). Scale bar: 30 μm. The proportions of follicles with different combinations of at least one BrdU^+^ and/or PCNA^+^ granulosa cell or completely BrdU^−^ and/or PCNA^−^ are expressed as a percentage of all follicles (**C**). An index difference was generated by subtracting the BrdU-labeling index from the PCNA-labeling index from each follicle (**D**) such that more positive values represent follicles that have decreased proliferation over time and more negative values representing follicles that increased proliferation rates over time (*P* < 0.001, ANOVA with Tukey’s *B* post hoc test, groups that share the same superscript letters are not significantly different). Co-labeling with immunohistochemistry for BrdU (red) and PCNA (green) was used to survey all granulosa cells throughout whole primary follicles in ovaries from mice that had been euthanized 1 day after BrdU withdrawal (**F**, **G**). (**E**) The percentage of granulosa cells labeled with PCNA, BrdU, or both labels was determined for all granulosa cells in confocal Z-stacks from 10 primary follicles (*N* = 694 granulosa cells). (**H**) The number of BrdU^+^ and PCNA^+^ cells was plotted for each follicle with regression line and 95% confidence interval and the expected regression line if there was a 1:1 ratio of BrdU^+^ and PCNA^+^ granulosa cells. BrdU-labeling index or PCNA-labeling index plotted against follicle diameter at day 0 (I), day 1 (J), and day 3 (K) demonstrate that the primary follicles with labeling indexes of zero tended to be the smaller follicles. BrdU: bromodeoxyuridine, PCNA: proliferating-cell nuclear antigen.

To investigate whether granulosa cell proliferation rates within a primary follicle remain constant or change over time, adjacent sections of primordial follicles were examined by BrdU and PCNA immunohistochemistry (Figure 5A and B), which labels cells in G1 and S-phase [15]. In this analysis, at the day 0 timepoint (the end of the 48-h BrdU exposure period), 23% of follicles were BrdU^−^/PCNA^−^ and 52% of follicles were BrdU^+^/PCNA^+^ (Figure 5C). There were also 23% of follicles that were BrdU^−^/PCNA^+^, possibly because BrdU is only incorporated into DNA during S-phase, whereas PCNA is expressed during G1 and S-phase [15]. This suggests that BrdU^−^/PCNA^+^ are follicles that entered G1 toward the end of the 48-h BrdU exposure period but had not yet entered S-phase. Twenty-four hours after the BrdU was withdrawn, the proportion of BrdU^−^/PCNA^+^ follicles had increased to 48%, indicating that follicles that had no proliferating granulosa cells during the 48-h BrdU exposure period now had signs of proliferation (Figure 5C). The follicles that were BrdU^−^ all had diameters <30 μm (Figure 5I–K), indicating that granulosa cells are less likely to be proliferating in the period immediately after transitioning from the primordial to primary stage. By 3 days post-BrdU withdrawal, the proportion of BrdU^−^/PCNA^+^ primary follicles was greatly diminished but BrdU^+^/PCNA^+^ cells were more abundant.

Co-labeling for BrdU and PCNA with confocal microscopy was used to count all granulosa cells throughout the whole volume of 10 primary follicles at the 1-day post-BrdU withdrawal timepoint (Figure 5F and G). A large proportion of the granulosa cells (46%) had neither BrdU nor PCNA staining (Figure 5E). BrdU^+^/PCNA^+^ cells represented 21% of granulosa cells, but only 7% of follicles were BrdU^+^/PCNA^−^ indicating that most granulosa cells that were proliferating during the 48-h BrdU exposure had continued to proliferate 24 h later. The remaining 26% of granulosa cells were BrdU^−^/PCNA^+^, representing granulosa cells that had been quiescent during the 48-h BrdU exposure but then had begun to proliferate in the following 24 h. The PCNA^+^ granulosa cell count was higher than the BrdU^+^ granulosa cell count in 8/10 follicles (Figure 5H), indicating that granulosa cell proliferation rates tended to increase over time in the primary follicles.

## Discussion

Granulosa cell proliferation occurs slowly in primary follicles, as many follicles had no visible BrdU^+^ granulosa cells in the histological cross-section after the 48-h BrdU exposure period. After the BrdU was withdrawn, the BrdU-labeled primary follicles progressed through to the secondary stage which allowed the duration of the primary follicle phase to be estimated at 14–16 days. It was not possible to replicate this pattern of follicle progression using models assuming constant rates of granulosa cell proliferation. Primary follicles were shown to have increasing proportions of proliferative granulosa cells over time and granulosa cells that had been through S-phase at least once during the 48-h BrdU period were more likely to be PCNA^+^ 24 h later. This indicates that primary follicles do not develop at constant rates and instead have periods with limited growth followed and periods of high proliferation.

Granulosa cell proliferation can be observed as soon as the primordial–primary follicle transition is initiated suggesting that this is one of the early events occurring in primordial follicle activation. One of the disadvantages of tritiated thymidine labeling or BrdU injections has been the low proportion of cells that take up the label in small follicles [3, 11]. Zheng et al. [7] used a transgenic approach were the *Sohlh1* gene promoter was used to activate a reporter gene in primordial follicle oocytes at a specific time, which resolves the issues associated with low uptake of label. Primary follicles with labeled oocytes were first observed 7 days after the reporter was induced and secondary follicles appeared after 23 days [7]. However, the authors used a previously described follicle classification [16], but the paper [7] uses a different nomenclature to describe their preantral follicles. Our best interpretation is that 7 days was required for primordial follicles to activate and develop to a state that we would classify as mid-primary, but the definition used to differentiate the onset of the secondary follicle phase remains unclear.

There is no consensus on preantral follicle stage classifications, as many authors choose bespoke classifications suitable for their study design. The classification that Pedersen and Peters [16] proposed in 1968 is often used, which classifies follicles based on the number of granulosa cell numbers per cross-section, but not biologically relevant changes in follicle function. In the present study, follicles that started to form the second layer of granulosa cells had a lower regression coefficient between oocyte diameter and granulosa cell number and labeling indexes never fell below 25% from this point forward. Therefore, the point of acquisition of the first granulosa cells in the second layer appears to represent a functional change that would be a suitable division between the primary and secondary follicle stages. Future gene expression experiments will be able to determine if this is the case.

In a preliminary assessment, total human follicle development time was estimated to be >355 days, with >150 days attributed to primary follicle phase and 120 days to the secondary phase [17], but no source was cited for the primary follicle estimate. The model was updated a decade later with the primary follicle development time removed [18], but the original >150-day primary follicle duration continues to be cited in recent literature [19, 20]. It is not clear if human primary or secondary follicles show variation in proliferation rates over time, but if so, the present study demonstrates that the prior methods used to calculate the duration of follicle development stages may be invalid. The duration of human follicle development is of renewed interest due to recent attempts to grow human follicles in vitro. Human primary follicles have been grown to 150 μm in diameter in as little as 8 days [21] or to 220 μm in 21 days [22]. This, coupled with the lack of experimental data to support the estimated 150-day primary follicle duration, suggests that the total duration of folliculogenesis may be substantially shorter than the often-cited >355-day estimate [17].

The design of the present study succeeded at determining the duration of the primary follicle phase, but it has several limitations. First, it remains possible that there is a population of very slow-growing follicles that take longer than 14 days to progress to the secondary stage and would also be less likely to take up any BrdU in the 48-h exposure period. Studies in rats have shown that 7 days of continuous infusion with [^3^H]thymidine is not sufficient to label all small primary follicles [23]. Second, it is not clear if follicles growing quickly or slowly have different susceptibilities to atresia. Third, the experiment was designed to determine the duration of the primary follicle phase but was not designed to establish the duration of follicle pauses or changes in the rates of proliferation within individual follicles. Fourth, the 48-h BrdU-exposure period was required to label a substantial proportion of primary follicles, but it is not clear if the last primary follicles to progress to the secondary phase were labeled at the beginning or the end of the exposure period. Despite these limitations, the experiment was able to determine the maximum duration of the primary follicle transition (14~16 days) and was able to demonstrate that granulosa proliferation rates within each primary follicle vary over time.

## Conclusion

This study demonstrates that there are distinct phases of granulosa cell proliferation in early preantral follicles and that the rate of growth is not constant. The two distinct phases of primary follicle development could be useful criteria for primary follicle classifications that represent distinct biological differences in function. Future work will need to determine if similar phases exist in other species, such as humans or agricultural species where assisted reproduction is commonly used.

## Data Availability

The data underlying this article will be shared on reasonable request to the corresponding author.
